# Astrocytes and retrograde degeneration of nigrostriatal dopaminergic neurons in Parkinson’s disease: removing axonal debris

**DOI:** 10.1186/s40035-021-00262-1

**Published:** 2021-11-02

**Authors:** Ingrid Morales, Ricardo Puertas-Avendaño, Alberto Sanchez, Adrian Perez-Barreto, Clara Rodriguez-Sabate, Manuel Rodriguez

**Affiliations:** 1 Laboratory of Neurobiology and Experimental Neurology, Department of Basic Medical Sciences, Faculty of Medicine, La Laguna University, La Laguna, Tenerife, Canary Islands Spain; 2 Center for Networked Biomedical Research on Neurodegenerative Diseases (CIBERNED) , Madrid, Spain

**Keywords:** Astrocyte, Phagocytosis, Parkinson’s disease, Dying-back degeneration, Medial forebrain bundle, Spheroid

## Abstract

**Objective:**

The dopaminergic nigrostriatal neurons (DA cells) in healthy people present a slow degeneration with aging, which produces cellular debris throughout life. About 2%–5% of people present rapid cell degeneration of more than 50% of DA cells, which produces Parkinson’s disease (PD). Neuroinflammation accelerates the cell degeneration and may be critical for the transition between the slow physiological and the rapid pathological degeneration of DA cells, particularly when it activates microglial cells of the medial forebrain bundle near dopaminergic axons. As synaptic debris produced by DA cell degeneration may trigger the parkinsonian neuroinflammation, this study investigated the removal of axonal debris produced by retrograde degeneration of DA cells, paying particular attention to the relative roles of astrocytes and microglia.

**Methods:**

Rats and mice were injected in the lateral ventricles with 6-hydroxydopamine, inducing a degeneration of dopaminergic synapses in the striatum which was not accompanied by non-selective tissue damage, microgliosis or neuroinflammation. The possible retrograde degeneration of dopaminergic axons, and the production and metabolization of DA-cell debris were studied with immunohistochemical methods and analyzed in confocal and electron microscopy images.

**Results:**

The selective degeneration of dopaminergic synapses in the striatum was followed by a retrograde degeneration of dopaminergic axons whose debris was found within spheroids of the medial forebrain bundle. These spheroids retained mitochondria and most (e.g., tyrosine hydroxylase, the dopamine transporter protein, and amyloid precursor protein) but not all (e.g., α-synuclein) proteins of the degenerating dopaminergic axons. Spheroids showed initial (autophagosomes) but not late (lysosomes) components of autophagy (incomplete autophagy). These spheroids were penetrated by astrocytic processes of the medial forebrain bundle, which provided the lysosomes needed to continue the degradation of dopaminergic debris. Finally, dopaminergic proteins were observed in the cell somata of astrocytes. No microgliosis or microglial phagocytosis of debris was observed in the medial forebrain bundle during the retrograde degeneration of dopaminergic axons.

**Conclusions:**

The present data suggest a physiological role of astrocytic phagocytosis of axonal debris for the medial forebrain bundle astrocytes, which may prevent the activation of microglia and the spread of retrograde axonal degeneration in PD.

**Supplementary Information:**

The online version contains supplementary material available at 10.1186/s40035-021-00262-1.

## Background

Available evidence suggests that the degeneration of the nigrostriatal dopaminergic neuron (DA cell) in Parkinson’s disease (PD) starts in the striatal synapse, and progresses retrogradely through the axons of the medial forebrain bundle (MFB) until reaching the cell soma in the substantia nigra (SN) [1]. DA cells have a thin (~ 0.5 µm), unmyelinated, and highly arborized (4 m total length and up to 1 million synapses in the human striatum) axon that supports high traffic of proteins and organelles and needs high levels of energy [2]. Although these characteristics make the dopaminergic (DAergic) axon particularly vulnerable to damage, their relevance for the retrograde degeneration of DA cells in PD is still little known. The DA cells of healthy people present slow degeneration with aging (6%–8% cell loss every decade), which produces cellular debris throughout life [3]. In the striatum, this debris may be removed by astrocytes without the help of microglia [4, 5], and the ability of synapses to counteract the DAergic denervation means that the injury goes unnoticed in most people. However, 2%–5% of people lose more than 50% of DA cells and present typical motor disorders of PD [3, 6]. These patients invariably present microglial activation and neuroinflammation in the striatum and SN [7]. Neuroinflammation accelerates the cell degeneration and may be critical for the transition between the physiological slow and the pathological quick degeneration of DA cells, particularly when it involves microglia of the MFB, which is near DAergic axons [8–11].

Synaptic debris produced from aging-related degeneration of DA cells may be removed by striatal astrocytes [4, 5], but the removal of axonal debris produced in the MFB during the retrograde degeneration of DA cells has been rarely studied [12]. The aim of this work was to study the removal of the axonal debris produced through retrograde degeneration of DA cells, paying particular attention to the relative roles of astrocytes and microglia. DAergic toxics (6-hyroxydopamine; 6-OHDA) injected in the striatal tissue can induce retrograde degeneration of DA cells, accompanied by microglial activation along the axon [13, 14]. There is evidence suggesting that this microglial activation is induced by the unspecific damage of striatal tissue (secondary to the toxicity of high-concentration 6-OHDA around the needle tip and to the physical damage generated by the needle penetration) more than that by the DAergic denervation itself [15]. Microglia activated in the striatum follow the axonal degeneration back across the MFB until reaching the SN, where they promote the degeneration of the DA cell somata (retrograde microgliosis) [1, 16–21]. We have reported a procedure that can induce selective DAergic denervation of the striatum not accompanied by unspecific damage to the striatal tissue and microgliosis [7]. This selective denervation stimulates the phagocytic activity of local astrocytes [7] which, without the collaboration of microglia, removes the protein [4] and organelle [5] debris produced from synaptic degeneration. The aim of the present work was to study whether the selective degeneration of DAergic striatal synapses is followed by retrograde degeneration of DAergic axons, and if the debris produced by this retrograde degeneration may be withdrawn by local astrocytes or with the help of microglial phagocytosis and neuroinflammation. These studies may help to understand the role of astrocyte phagocytosis as a procedure to prevent retrograde microgliosis and neuroinflammation in healthy people [22, 23].

## Methods

### Animals and lesions

Experiments were carried out in 40 male Sprague–Dawley rats weighing 300–350 g and in YFP-Mito-DAn mice (25–30 g). The YFP-Mito-DAn mouse was generated by crossing mice which express a yellow fluorescent protein (YFP) targeted to the mitochondrial matrix but which have a lox-flanked stop cassette placed upstream of the mito-YFP transgene to restrict the expression of the protein to cells in which the stop cassette had been removed by cre-mediated excision (mice generously provided by Prof. Nils-Göran Larsson; Max-Planck Institute for Biology of Ageing; Köln; Deutschland) [24], with DAT-cre mice expressing cre under control of the dopamine transporter (DAT) (B6.SJL-Slc6a3^tm1.1(cre)Bkmn^/J; The Jackson Laboratory, Bar Harbor, ME). The resulting offspring had fluorescent mitochondria in DAergic neurons (YFP-Mito-DAn), rather than in other cells of the brain [5]. Animals were housed at 22 °C, two per cage, under normal laboratory conditions under a standard light–dark schedule with free access to food and water.

The animals were anaesthetised with ketamine (25–40 mg/kg i.p. in rats and 25–40 mg/kg i.p. in mice; Rhône Mérieux; Lyon, France) and xylazine (3–6 mg/kg i.p. in rats and 25–40 mg/kg i.p. in mice; Bayer, Leverkusen, Germany), and injected (1 µl/min) in the lateral ventricle (Kopf Instruments, Tujunga, CA; coordinates: 1.4 mm lateral to the midline, 0.8 mm posterior to bregma and 4 mm below the dura in rats and 1.4 mm lateral to the midline, 0.8 mm posterior to bregma and 4 mm below the dura in mice) with vehicle (0.3 µg/µl ascorbic acid in 0.9% saline) or a single dose of 6-OHDA (Sigma, St. Louis, MO) (25 µg in 10 µl saline solution in rats and 5 µg in 5 µl in mice). In order to prevent degeneration of noradrenergic cells, the noradrenaline uptake was inhibited with nortriptyline (Sigma, St. Louis, MO; 30 mg/kg injected i.p. 20 min before 6-OHDA administration).

### Tissue processing

The rats were anaesthetized with chloral hydrate (400 mg/kg i.p.) and, after checking for the complete anaesthetic state (e.g. loss of corneal reflex by lack of blinking when air is blown into eyes and loss of pedal pain reflex by lack of movement of paw/tail when squeezed), they were transcardially perfused with 200 ml of 0.9% saline solution followed by 400 ml of 4% paraformaldehyde in 0.1 M phosphate buffer (PBS) pH 7.4 at 4 h, 1 day and 5 days after 6-OHDA administration [25]. The brains were removed and stored in the same fixative at 4 ºC for 4 h, immersed in a cryoprotective solution of 30% sucrose in the same buffer for 48 h and then cut at 30 μm with a sliding microtome (HM 450, MICROM International GmbH; Walldorf, Baden-Wurttemberg, Germany) following axial planes parallel to the surface of the brain cortex and perpendicular to the probe trajectory. Sections were collected in 7 parallel series and processed for immunohistochemistry [26].

### Immunofluorescent labelling

For immunofluorescent labeling, floating sections were first incubated for 1 h at room temperature (RT) in 4% normal goat serum (NGS, Sigma-Aldrich, Madrid, Spain) in PBS and 0.05% Triton X-100 (TX-100, Sigma-Aldrich, Madrid, Spain), and overnight in the same solution containing the primary antibodies shown in Table 1. Finally, triple and quadruple immunofluorescent labeled sections were incubated for 2 h with the secondary antibodies in PBS containing 1:200 NGS followed by Alexa Fluor 488-, CY3-, or DyLight 649-conjugated streptavidine (Jackson ImmunoResearch, Bar Harbor, ME) in PBS.

**Table 1:** Antibodies used in the study

Antibody	Dilution	Host	Producer, catalogue number
*Primary antibody*			
APP	1:1500	Rabbit	Abcam, ab32136
CD68	1:1000	Mouse	AbD Serotec, MCA341GA
Cathepsin	1:100	Goat	St John´s Lab, STJ140018
DAT	1:50	Goat	Santa Cruz Biotechnology,Inc.,sc1433
GDNF	1:500	Rabbit	Alomone, ANT-014
GFAP	1:1000	Mouse	Millipore, MAB360
GFAP	1:1000	Rabbit	Sigma, G9269
GFP (YFP)	1:1000	Rabbit	Abcam, ab6556
IBA1	1:500	Rabbit	Wako, 019-19741
IBA1	1:500	Mouse	Millipore, MABN92
LAMP1	1:100	Mouse	Abcam, ab25630
LAMP2	1:100	Goat	Santa Cruz Biotechnology, Inc., sc8100
LC3-II	1:1200	Rabbit	Abcam, ab48394
LC3-II	1:200	Goat	Santa Cruz Biotechnology, Inc., sc16755
P62 (SQSTM1)	1:400	Guinea Pig	Origene, BP5002
SYN	1:200	Rabbit	Sigma, S3062; Millippore, AB5038
TH	1:1200	Chicken	Abcam, ab76442
VMaT2	1:1500	Guinea Pig	Origene, EUD2801
YFP	1:200	Goat	St Johnsons lab, STJ140118
*Secondary antibody*			
AF 488 anti-Chicken	1:400	Donkey	Jackson ImmunoResearch, 703-545-155
AF 647 anti-Chicken	1:400	Donkey	Jackson ImmunoResearch, 703-605-155
AF 488 anti-Goat	1:400	Donkey	Jackson ImmunoResearch, 705-545-147
AF 647 anti-Mouse	1:400	Donkey	Jackson ImmunoResearch, 715-605-151
Biotin anti-Mouse	1:300	Donkey	Jackson ImmunoResearch, 715-065-151
Cy3 anti-Rabbit	1:400	Donkey	Jackson ImmunoResearch, 711-165-152
Biotin anti-Rabbit	1:1000	Donkey	Jackson ImmunoResearch, 711-065-152
AF 488 anti-Guinea Pig	1:1000	Goat	Jackson ImmunoResearch, 106-545-003
Biotin anti-Guinea Pig	1:1000	Goat	Jackson ImmunoResearch, 106-065-003
AF 488 anti-Chicken	1:800	Goat	Invitrogen, A11039
AF 546 anti-Chicken	1:600	Goat	Invitrogen, A11040
AF 546 anti-Mouse	1:1000	Goat	Invitrogen, A21123
AF 488 anti-Mouse	1:400	Goat	Jackson ImmunoResearch, 115-546-146
Biotin anti-Mouse	1:1200	Goat	Jackson ImmunoResearch, 115-065-003
Cy3 anti-Rabbit	1:500	Goat	Jackson ImmunoResearch, 111-165-144
Biotin anti-rabbit	1:1200	Goat	Jackson ImmunoResearch, 111-065-144
*Others*			
405-streptavidin	1:400	–	Jackson ImmunoResearch, 016-470-084
Dylight 649-streptavidin	1:600	–	Jackson ImmunoResearch, 016-490-084
488-streptavidin	1:800	–	Jackson ImmunoResearch, 016-540-084
Cy3-streptavidin	1:600	-	Jackson ImmunoResearch, 016–160-084

TH: tyrosine hydroxylase, DAT: dopamine transporter, GFAP: glial fibrillary acidic protein, App: amyloid precursor protein, Syn: α-synuclein, GDNF: glial-derived neurotrophic factor, Lamp1: lysosomal-associated membrane protein 1, Lamp2: lysosomal-associated membrane protein 2, CD68: cluster of differentiation 68, LC3: microtubule-associated protein 1A/1B-light chain 3, VMat2: vesicular monoamine transporter 2, Iba1: ionized calcium binding adaptor molecule 1, YFP: yellow fluorescent protein

After several rinses, the sections were mounted on gelatinized slides, air dried, coverslipped with Vectashield (Vector, Burlingame, CA), and examined under a confocal microscope (Olympus Fluoview FV1000 and Leica SP8) using appropriate filters. Appropriate controls were performed to test the immunohistochemical assays. Primary antibodies were selected because they had been previously tested in different studies and in many brain sites including the striatum, and because the selectivity for their biomolecular targets was confirmed by western blot and absorption controls. Before applying primary antibodies, the tissues were inspected under bright-field and fluorescence microscope to ensure that the endogenous tissue background was not interfering with the study. Controls with the tissue incubated without the primary antibody but including the secondary antibody were also performed. When necessary, control studies were also performed where the primary antibody was replaced by a specific isotype (Chicken IgY isotype control, AB-101-C, Novus Biologicals, Centennial, CO; Goat IgG isotype control, AB-108-C, Novus Biologicals; Rabbit IgG monoclonal [EPR25A] isotype control, ab 172730, Abcam, UK; Mouse IgG1 isotype control, NBP1-97005, Novus Biologicals; Rabbit IgG isotype control, NBP2-21952, Novus Biologicals). During the initial optimization studies, all antibodies were tested in tissue samples or cells that are known to express the epitope of interest. These control tests were performed during the initial optimization studies and, when possible, they were also performed in the same tissue samples (looking for other cells or sub-cellular components which are known to present the epitope of interest). Quantitative analysis was performed with the ImageJ (IJ1.46r; Bethesda, MD) software.

### Quantitative analysis and statistical comparisons

The diameters of the axons and spheroids were computed in the MFB ipsilateral to the 6-OHDA administration as previously reported [7, 27]. Quantitative studies and analysis of the distribution of fluorescent signals were always performed on single-plane images and not on 3D-z stack images. The immunoreactivity of proteins was evaluated by densitometric analysis of DAergic axons and spheroids, astrocytic processes and astrocytic soma. Densitometric data were normalized as a percentage of the mean value of extracellular locations. In order to prevent differences arising from variations in protocol conditions during tissue processing and densitometric analysis, all sections were processed simultaneously using the same protocols and chemical reagents, and all microscopic and computer parameters were kept constant throughout the densitometric study. Measurements were performed with the FV10-ASW (version 01.07.01.00; Olympus Iberia, H’Hospitalet de Llobregat, Spain) and ImageJ (IJ 1.46r) softwares. Subcellular colocalizations were performed with the Leica Application Suite Advanced Fluorescence, and with the Colocalization, RG2B colocalization, Colocalization_Finder and JACoP plugins of the ImageJ program [28, 29].

### Study of axons and spheroids by electron microscopy (EM): immunolocalisation of DAT and TH

Eight male mice, four WT (C57BL/6) and four YFP-Mito-DAn, were deeply anaesthetised with a mixture of ketamine (100 mg/kg; Parke-Davis, Alcobendas, Spain) and 2% xylazine (10 mg/kg; Dibapa, Barcelona, Spain), and fixed by intracardiac perfusion of Sorensen`s phosphate buffer (0.1 M, pH 7.4) containing 4% paraformaldehyde and 0.05% glutaraldehyde. The mice were then decapitated and their striatal tissues quickly removed and postfixed by immersion in the same fixative for 1 h. Then the tissues were processed for double-labelling using the pre-embedding HRP/immunogold method. Free‐floating sections obtained from the two genotypes (WT and YFP-Mito-DAn) were incubated in parallel in 10% (*v*/*v*) NGS diluted in tris buffered saline (TBS). The sections were then incubated in rat anti‐DAT and rabbit anti-TH antibodies (3–5 μg/ml diluted in TBS containing 1% (*v*/*v*) NGS), followed by incubation with a mixture of goat anti‐rat IgG coupled to 1.4-nm gold (Nanoprobes Inc., Stony Brook, NY) and anti-rabbit IgG biotinilate. The sections were postfixed in 1% (*v*/*v*) glutaraldehyde and washed in double‐distilled water, followed by silver enhancement of the gold particles with an HQ Silver kit (Nanoprobes Inc., Gentaur France SARL, Paris, France). Then they were incubated in 0.05% 3–3′diaminobenzidine tetrahydrochlorhidre, treated with osmium tetraoxide (1% in 0.1 M phosphate buffer), block‐stained with uranyl acetate, dehydrated in graded series of ethanol and flat‐embedded on glass slides in Durcupan (Fluka) resin. Regions of interest were cut at 70–90 nm on an ultramicrotome (Reichert Ultracut E, Leica, Austria) and collected on single slot pioloform-coated copper grids. Staining was performed on drops of 1% aqueous uranyl acetate followed by Reynolds's lead citrate. Ultrastructural analyses were performed with a JEOL‐1010 electron microscope (0.4 nm resolution) equipped with a W filament (Japan), a Gatan Orius SC200W 1 camera, and GMS DigitalMicrograph software version 2.31 (2014). The specificity of the immunocytochemistry was tested by replacing the different antiserums with normal serum.

### Statistical analyses

Colocalizations were quantified with Pearson’s correlation coefficient, the cytofluorogram, the Manders overlap coefficients M1 (fraction of the marker 1 overlapping with marker 2) and M2 (fraction of the marker 2 overlapping with marker 1), the colocalization rate (the extent of colocalization in percentages), and the overlap coefficient (a value between 0 and 1—the closer the value is to 1, the stronger the colocalization). Sample comparisons were performed with the Scheffé test using the statistic program Statsoft (Tulsa, OK). *P* < 0.001 was considered as statistically significant.

## Results

### Selective damage to DAergic synapses in striatum induces retrograde degeneration of DAergic axons

**Fig. 1:**
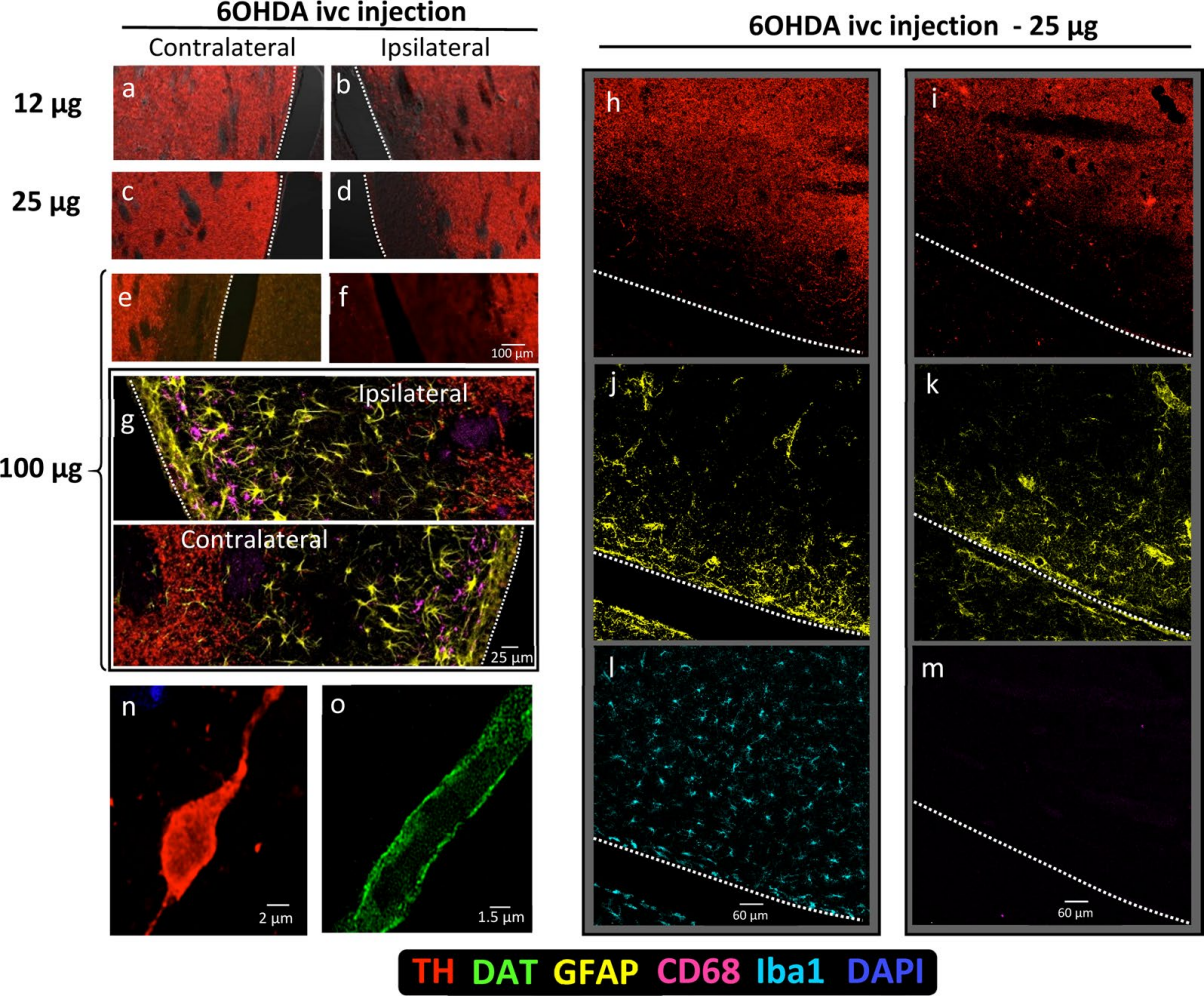
Selective degeneration of DAergic terminals in the striatum. **a**–**f** An example of the effect of increasing doses of 6-OHDA administered in the lateral ventricle 5 days before the study. **g** An example showing that the highest 6-OHDA dose (100 µg) induces astrocytosis and microgliosis in both the ipsilateral (**g** top) and contralateral (**g** bottom) striatum. The 25 µg dose denervated the nearby tissue of the ipsilateral but not of the contralateral striatum (**c** vs **d, h**, **i**), inducing local astrocytosis (**j**, **k**), with no change in the number of microglial cells (Iba1 in **l**) and with no macrophage activation (CD68 in **m**). The DAergic denervation was accompanied by thickening of axons, which facilitated the formation of spheroids (**n**). **o** A thickened axon whose membrane was marked with a DAT antibody

The 6-OHDA perfusion in the lateral ventricle caused degeneration of the DAergic synaptic terminals in areas of the ipsilateral striatum closest to the ventricle. 6-OHDA (12–100 µg) induced dose-dependent DAergic denervation of the ipsilateral striatum adjacent to the lateral ventricle, five days after administration (Fig. 1b, d, f). Although 12 µg and 25 µg of 6-OHDA only induced a local unilateral effect, the highest dose (100 µg) also denervated the contralateral striatum (Fig. 1a, c, e). The denervated areas showed astrocytosis but not microgliosis. This astrocytosis increased from 24 h to five days and persisted for at least three weeks. Figure 1 right panels show two examples of this astrocytosis (Fig. 1j, k) 24 h after striatal denervation with 25 µg of 6-OHDA (Fig. 1h, i). No changes in the number of microglial cells (Fig. 1l) and no microglial activation (CD68 in Fig. 1m) were observed in the striatal areas denervated with low doses of 6-OHDA (25 µg). However, the high dose of 6-OHDA (100 µg), which induced contralateral denervation of the striatum (Fig. 1e), also induced macrophagic activation of microglia in the striatal areas near the ipsilateral (Fig. 1g top) and contralateral (Fig. 1g bottom) ventricle. Thus, the 6-OHDA doses used in this study were those that induced selective DAergic denervation and selective astrocyte activation (not accompanied by microglia activation) in the striatum of the same side of 6-OHDA perfusion (25 µg). A similar study was performed in mice in which the 6-OHDA dose selected (12.5 µg) induced unilateral selective denervation of the striatum that was accompanied by astrocytosis but not by microgliosis.

In both rats and mice, the denervation was accompanied by thickening of the surviving axons of the striatum that later generated a beading of the axonal structure, producing spheroids (Fig. 1n for an axon that is producing a spheroid, Fig. 1o for a thickened axon [> 2 µm diameter] with DAT immunoreactivity mainly located in the axonal membrane). Twenty-four hours after 6-OHDA administration, the thickened axons (> 2 µm diameter) and spheroids appeared in the posterior areas of the striatum and anterior areas of the MFB. Five days after 6-OHDA administration, the degenerating axons and the corresponding spheroids were identified in the medial regions of the MFB which was located lateral to the incerto-hypothalamic DAergic cells of the hypothalamus. These findings suggest a rostro-caudal movement of axonal degeneration starting four hours after the toxic administration, and progressing to posterior regions during the five days of the study. No microgliosis and no clear evidence of astrocytosis were found in the MFB 24 h and 5 days after 6-OHDA administration.

**Fig. 2:**
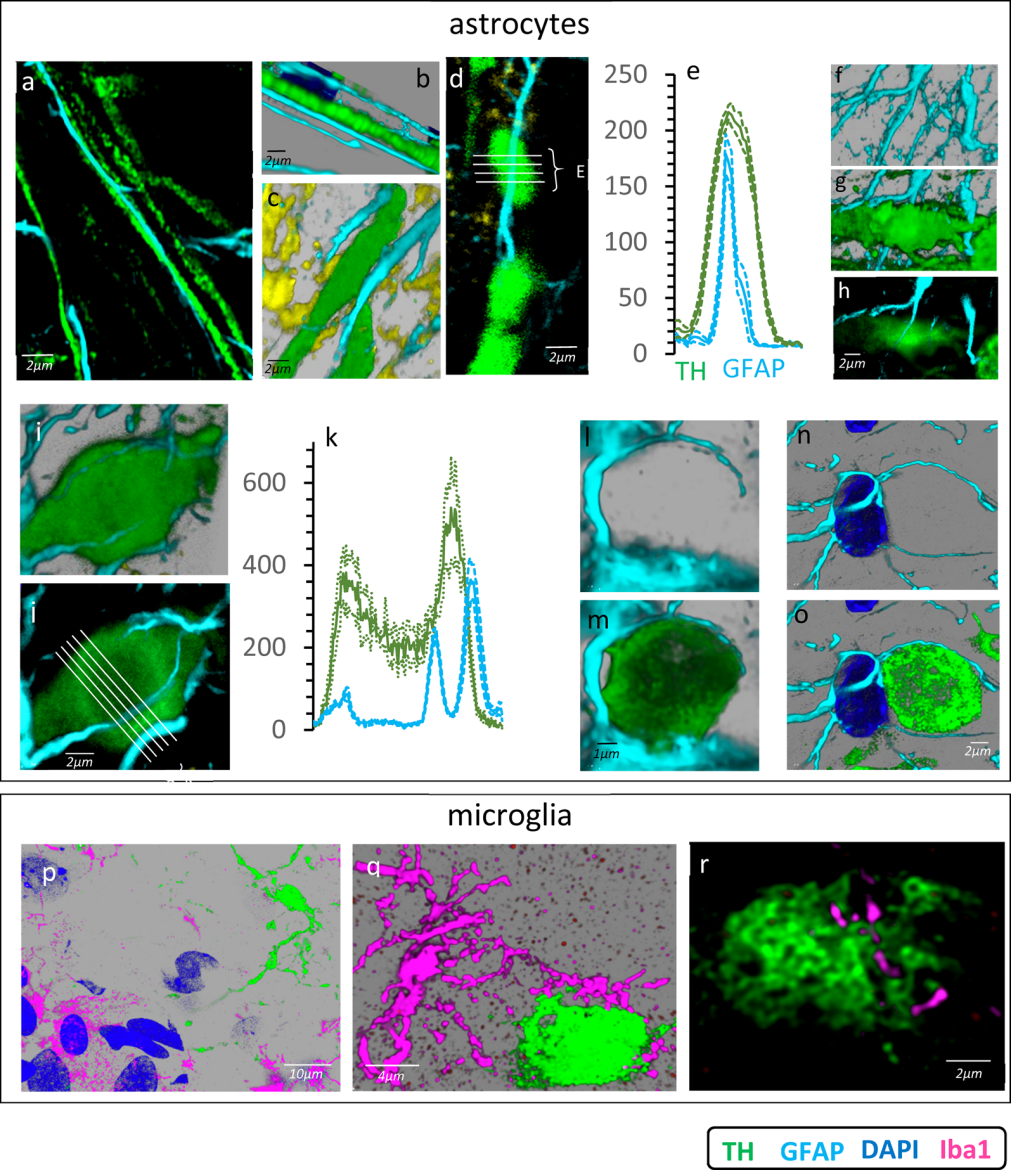
Retrograde degeneration of DAergic axons in the MFB. **a**, **b** Normal DAergic thick axons and astrocytic processes of the MFB. **c** A thick degenerating axon surrounded by astrocytic processes. **d** Three spheroids produced by fracture of a degenerating axon (TH in green) and which were penetrated by an astrocytic process (cyan). **e** Mean ± standard error of fluorescence values under the white lines shown in **d**. **f**, **g** A spheroid penetrated by an astrocytic process (slice through which the astrocytic process passed is shown in **h**). **i** A spheroid penetrated by two astrocytic processes. **j** A slice of the spheroid in **i**. **k** The mean ± standard error of white lines shown in **j**. **l**, **m** A spheroid surrounded by an astrocytic process. **n**, **o** A spheroid near the cell somata of an astrocyte and surrounded by two astrocytic processes of this cell. **p** A spheroid (top-right) and distant microglial cells (bottom-left). **q** A microglial cell with projections near a spheroid. **r** A slice of a spheroid with nearby small processes of a microglial cell

The healthy axons of the MFB were accompanied by thin astrocytic processes that followed a parallel course very close to them (Fig. 2a). The thick DAergic axons undergoing retrograde degeneration showed a similar relationship with astrocytic processes (Fig. 2b, c; Additional file 1: Video 1). The degenerating axons of the MFB progressively increased their diameter (from 0.5 to 2–6 µm) and split along their main axis to form spheroids (Fig. 2d), which were then penetrated by the nearest astrocytic process (Figs. 2f–h; Additional file 2: Video 2). Figure 2e shows the distribution of GFAP immunoreactivity (cyan) of an astrocyte process located inside the TH immunoreactivity (green) of the spheroid shown in Fig. 2d. Some large spheroids were both penetrated and surrounded by astrocytic processes. Figure 2k shows the distribution of GFAP immunoreactivity (cyan) of two astrocyte processes located near or inside the TH immunoreactivity (green) of the spheroid shown in Fig. 2i, j. Other spheroids (often located near the somata of astrocytes) were surrounded but not penetrated by astrocytic processes (see two examples in Fig. 2l–o). Additional file 3: Video 3 shows the spheroid of Fig. 2m, showing the internal low-density which is typically associated with the flocculation of spheroids. The microglia of the MFB were not generally located in regions close to degenerating axons or to spheroids (Fig. 2p), although some microglial cells showed prolongations that approached the spheroids (Fig. 2q) and that seemed to make direct contact with them (Fig. 2r).

### Proteins and organelles produced by the retrograde degeneration of DAergic axons are stored in spheroids

**Fig. 3:**
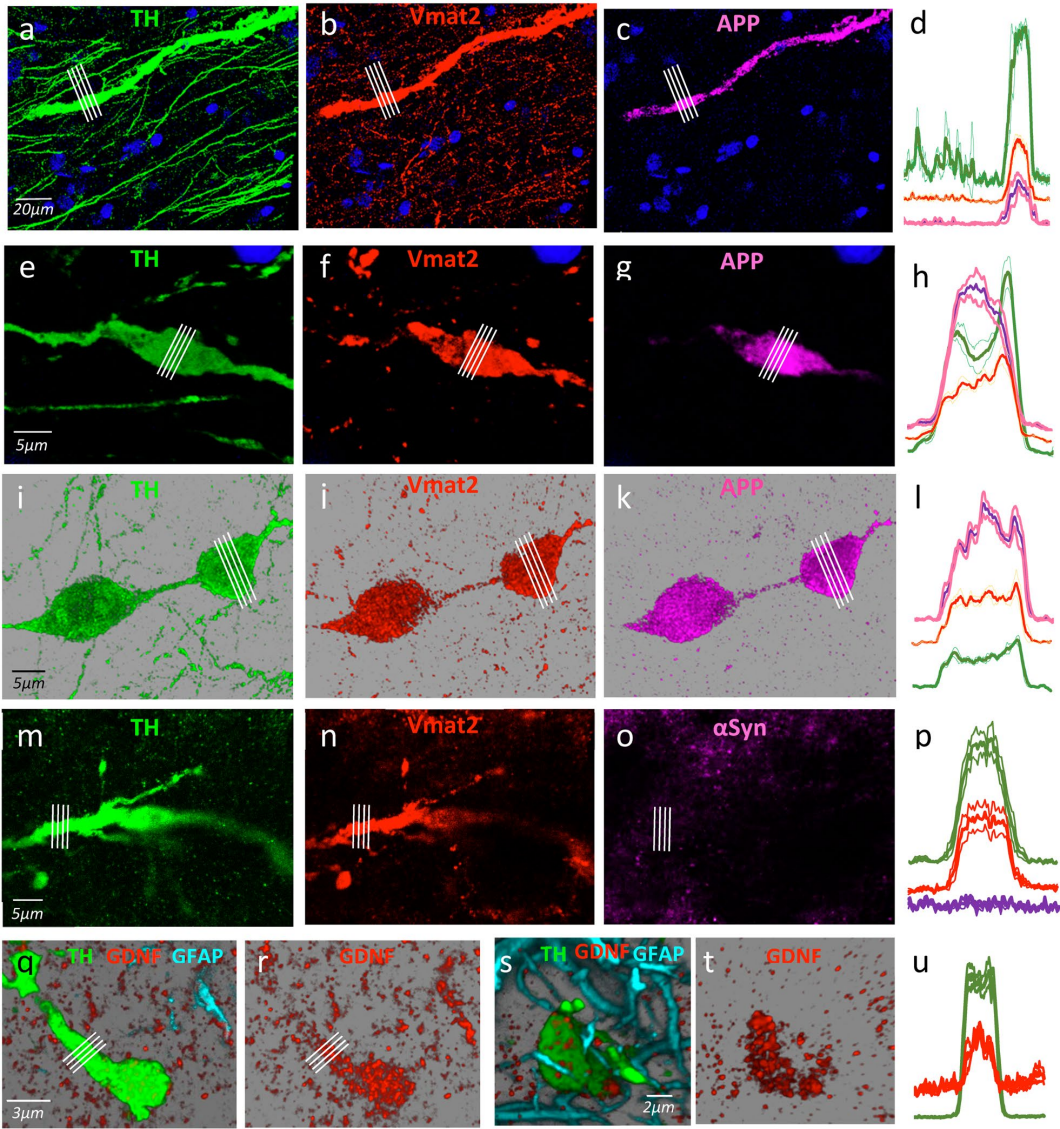
The degenerating axons and spheroids accumulate DAergic debris. **a** A thick degenerating axon between normal thin axons of the MFB. **b**, **c** Accumulation of Vmat2 and APP respectively in the thick axon but not in the thin axons. **d** The fluorescence values under the white lines of **a**–**c** (mean ± standard error). **f, g** Vmat2 and APP immunoreactivity in the spheroid shown in **e**. **h** The mean ± standard error of fluorescence values indicated by the white lines in **e**–**g**. **j**, **k** Vmat2 and APP immunoreactivity of the two spheroids that were being formed in **i** by the fracture of a DAergic axon. **l** The mean ± standard error of the fluorescence values indicated by the white lines in **e**–**g**. **n**, **o** Vmat2 and αsyn immunoreactivity of the thick degenerating axon shown in **m**. **p** The mean ± standard error of the fluorescence values indicated by the white lines in **m**–**o**. **r** The GDNF immunoreactivity of the spheroid shown in **q**. **t** The GDNF immunoreactivity of the spheroid shown in **s**. **u** The mean ± standard error of the fluorescence values indicated by the white lines in **q** and **r**

The DAergic spheroids of the MFB accumulated the proteins and organelles that otherwise normally move through the DAergic axons. Figure 3a shows an example of a thickened TH^+^ degenerating axon located between other normal TH-axons of the MFB. The thickened axons (but not the normal thin axons) showed a markedly increased immunoreactivity for Vmat2 and APP (Fig. 3b–d). A similar high immunoreactivity for TH and Vmat2 was found in the thickening process of axon that precedes the formation of spheroids (Fig. 3e–h), and also in the fully formed spheroids (Fig. 3i–l). Additional file 4: Video 4 shows a fully formed DAergic spheroid containing Vmat2 (red) and APP (pink). TH, Vmat2 and APP are synthesized in the nigral DA-cell somata, and then migrate along the axon until they reach the synaptic terminals in the striatum. The present findings suggest that proteins that cannot follow their anterograde migration along the degenerating axons are stored within spheroids, which prevents their release and dispersion through the extracellular medium. However, not all proteins with anterograde motion were stored in spheroids. In an example of a degenerating axon (Fig. 3m), Vmat2 (Fig. 3n) but not α-synuclein (αsyn) (Fig. 3o) was accumulated (Fig. 2p). Retrogradely transported proteins may also be stored in the spheroids formed from DAergic axons. Here we found that GDNF, a neurotrophic factor which is normally introduced into the DAergic synapses of the striatum, and then is retrogradely transported through the axon to the soma of the DA-cell in the SN, was accumulated in DAergic spheroids (TH^+^) (Fig. 3q–u, Additional file 5: Video 5).

**Fig. 4:**
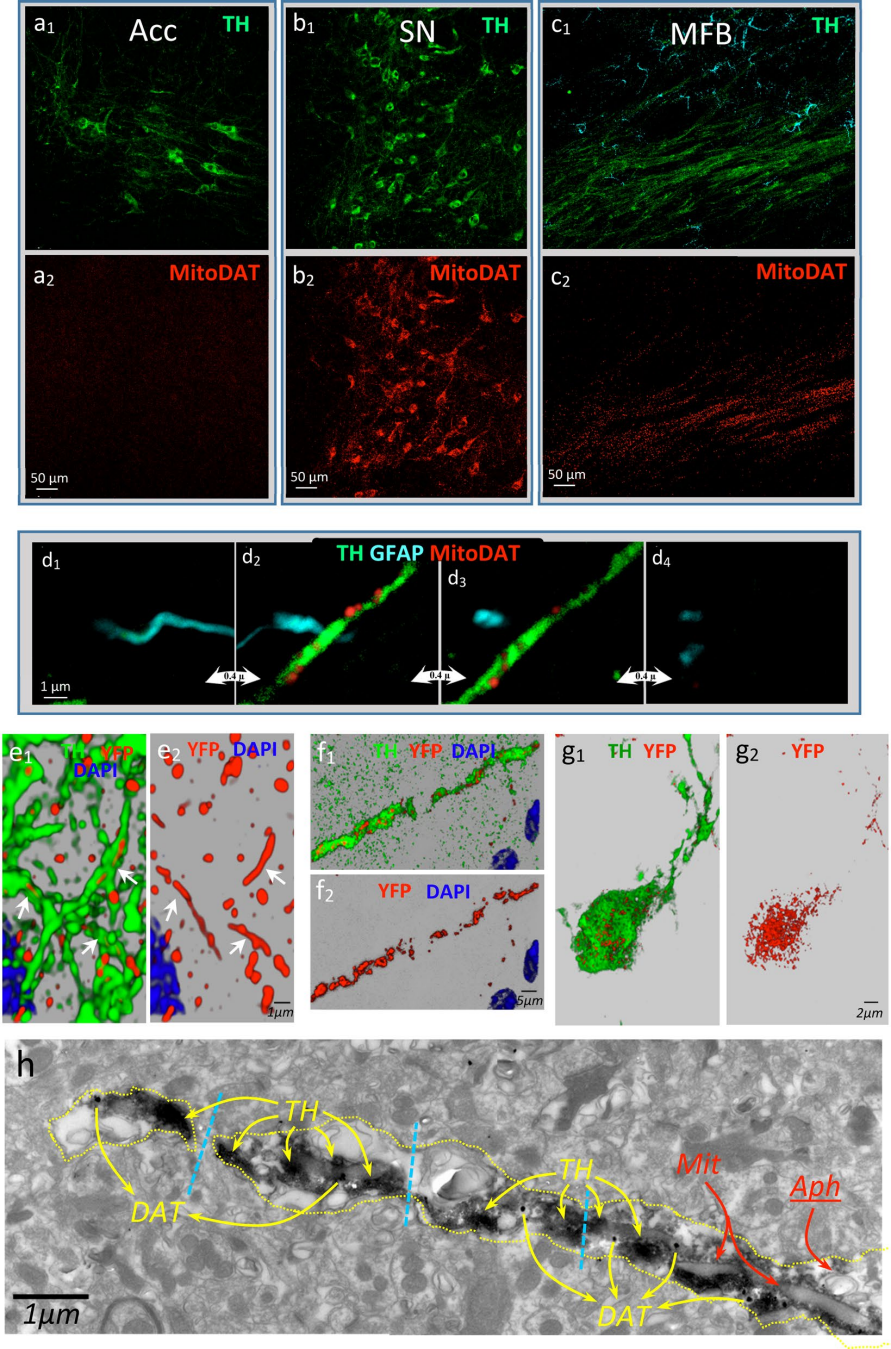
The degenerating axons and spheroids accumulate DAergic mitochondria. The fluorescent mitochondria were found in DAergic neurons but not in other brain cells in YFP-Mito-DAn mice. Noradrenergic neurons of the nucleus accumbens present TH (**a**_**1**_) but not YFP immunoreactivity (**a**_**2**_). DAergic nigrostriatal neurons present both TH and YFP immunoreactivity, a fact observed in their cell somata (**b**_**1**_, **b**_**2**_) and in their axons in the MFB (**c**_**1**_, **c**_**2**_). **d**_**1**_–**d**_**4**_ A normal DAergic axon of the MFB with fluorescent mitochondria, and a nearby astrocytic process with no fluorescent mitochondria (successive z-axis slices obtained 0.4 microns thick). **e**_**1**_, **e**_**2**_ Elongated mitochondria (white arrows) observed in DAergic axons of the normal striatal tissue. The retrograde damage of DAergic neurons produced degenerating axons in the MFB where the mitochondria were broken into small pieces (**f**_**1**_, **f**_**2**_). These mitochondria were finally retained within the spheroids (**g**_**1**_, **g**_**2**_). **h** An electron microscopy image showing a degenerating DAergic axon of the MFB with TH and DAT immunoreactivity and containing a long damaged mitochondria (Mit) and an autophagosome (Aph)

Not only proteins but also the organelles that normally move along the DAergic axons may be stored in spheroids. This was the case for mitochondria. The movement of mitochondria was studied using the YFP-Mito-DAn mice that present fluorescent mitochondria in DAergic neurons but not in other brain cells. Figure 4 top shows DAergic neurons of the SN (Fig. 4b_1_) whose somata present many fluorescent mitochondria (Fig. 4b_2_). These neurons also had many fluorescent mitochondria (Fig. 4c_2_) distributed along their axons (Fig. 4c_1_). These fluorescent mitochondria were not observed in other cells including monoaminergic neurons (Fig. 4a_1_, a_2_) and astrocytes (Fig. 4c_1_, c_2_). Figure 4d_1-4_ shows successive z-axis slices of a normal DAergic axon of the MFB with fluorescent mitochondria, and a nearby astrocytic process with no fluorescent mitochondria. Some axonal mitochondria showed a spheroid shape while others had an elongated shape that was prolonged by the interior of the axon (Fig. 4e_1_, e_2_, Additional file 6: Video 6). However, when the retrograde damage of DAergic neurons produced degenerating axons in the MFB, the mitochondria were broken into small pieces (Fig. 4f_1_, f_2_). These mitochondria were finally retained within spheroids (Fig. 4g_1_, g_2_), thus preventing their dispersion through the extracellular medium. EM image shows a degenerating DAergic axon (DAT^+^) that began to fracture into spheroids (discontinuous cyan lines) and contained elongated damaged mitochondria (Mit) and autophagosomes (Aph) (Fig. 4h).

### Mechanisms for protein and mitochondrial degradation are not present in the DAergic spheroids

**Fig. 5:**
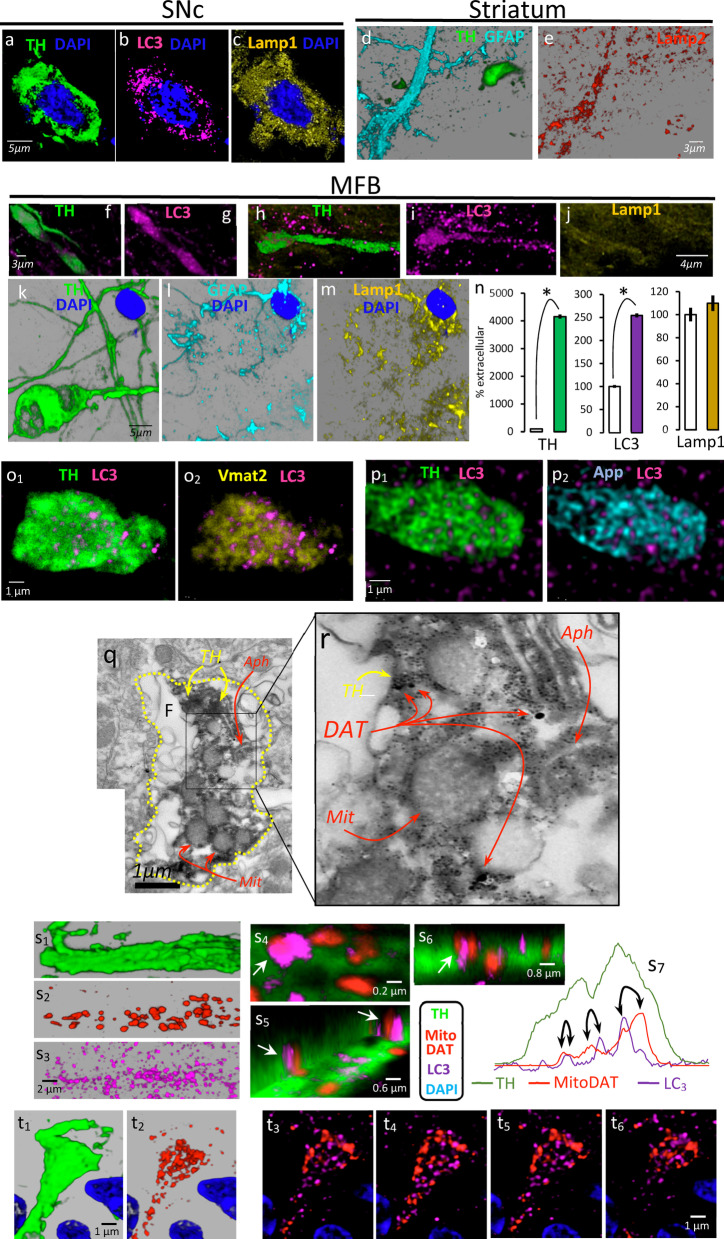
Dopaminergic autophagy begins in axons and spheroids. Top-left images show the somata of a nigral DAergic neuron with immunoreactivity for LC3 (autophagosomes) and Lamp1 (lysosomes). Top-right images show Lamp 2 (lysosomes) immunoreactivity (**e**) in a process of a striatal astrocyte (cyan in **d**) but not in a nearby spheroid (green in **d**). Bottom images were obtained in the MFB. **g** LC3 immunoreactivity in the degenerating axon shown in **f**. **i** An example of LC3 immunoreactivity found in a degenerating axon (**h**) that does not present Lamp1 immunoreactivity. **k**–**m** present Lamp1 immunoreactivity (**m**) in an astrocyte but not in a nearby spheroid (**k**). **n** shows the mean ± standard error of the density of the TH, LC3 and Lamp1 immunoreactivity (normalized as a percentage of the mean value of the extracellular medium) computed for MFB spheroids (*n* = 120; *P < 0.001). **o**_**1**_, **p**_**1**_ Two examples of DAergic spheroids that present a diffuse and generalized accumulation of Vmat2 (**o**_**2**_) and APP (**p**_**2**_) together with a puncta-like distribution of LC3 immunoreactivity. **q**, **r** Electron microscopy images of a DAergic spheroid delimited with yellow dotted lines (**q**), which presented flocculated areas (F) and high density areas with TH and DAT immunoreactivity, and contained damaged mitochondria (Mit) and autophagosomes (Aph). **s**_**1**_ An example of a degenerating thick axon which accumulated mitochondria (**s**_**2**_) and began to produce autophagosomes (**s**_**3**_). **s**_**4**_–**s**_**6**_ 3D images of a degenerating axon with some of their mitochondria near autophagosomes (white arrows), a fact also observed in spheroids. **s**_**7**_ Distribution of mitochondria (YFP fluorescence in the red line) and autophagosomes (puncta of LC3 fluorescence in the purple line) inside a typical DAergic spheroid (TH fluorescence in the green line). However, mitochondria began to break apart (**t**_**2**_) within the spheroids (**t**_**1**_), and not all mitochondria showed a clear link to autophagosomes at this time (**t**_**3**_–**t**_**6**_)

Nigral DA-cells (Fig. 5a) and striatal astrocytes (Fig. 5d) showed proteins involved in macro-autophagia, including LC3 of autophagosomes (Fig. 5b) and Lamp1 (Fig. 5c) and Lamp2 (Fig. 5e) of lysosomes. The thickened TH^+^ degenerating axons of the MFB (Fig. 5f, h) showed LC3 immunoreactivity (Fig. 5g) with characteristic puncta distribution of formations of autophagosomes (Fig. 5i). However, these axons did not show Lamp1/Lamp2 immunoreactivity (Fig. 5j). Figure 5k–m shows an example of astrocyte (Fig. 5l) with Lamp1 immunoreactivity (Fig. 5m) near a DAergic spheroid with no Lamp1 immunoreactivity (Fig. 5k). The spheroids showed a marked increase of TH immunoreactivity and a 150% increase of the LC3 immunoreactivity (versus the extracellular medium). However, no significant differences were found in the case of Lamp1 (Fig. 5n, right) or Lamp2 (data not shown). Figure 5o shows an example of a spheroid with TH (o_1_) and Vmat2 (o_2_) immunoreactivity and presenting a number of autophagosomes (puncta LC3 immunoreactivity). Similarly, Fig. 5p shows an example of a spheroid with TH (p_1_), APP (p_2_) and puncta LC3 immunoreactivity. The sparse distribution of TH, Vmat2 and APP immunoreactivity indicates that these proteins are scattered throughout the spheroid and not particularly linked to autophagosomes (see also Additional file 7: Video 7). EM image of an example of DAergic spheroid (TH^+^ in Fig. 5q and DAT in Fig. 5r) with autophagosomes (Aph) is also shown.

The degenerating DAergic axons (Fig. 5s_1_) with accumulation of mitochondria (Fig. 5s_2_) began to generate autophagosomes (Fig. 5s_3_), some of which were close or even linked to the mitochondria (white arrows in Fig. 5s_4_–s_6_). Figure 5s_7_ shows an example of close localization (black arrows) of mitochondria (YFP in red) and autophagosomes (puncta of LC3 in purple) inside a DAergic spheroid (TH in green). The mitochondria began to break apart (Fig. 5t_2_) in a spheroid (Fig. 5t_1_), and not all of them showed a clear link to autophagosomes at this time (Fig. 5t_3_–t_6_).

### Astrocytes but not microglia engulf the debris of DAergic spheroids

**Fig. 6:**
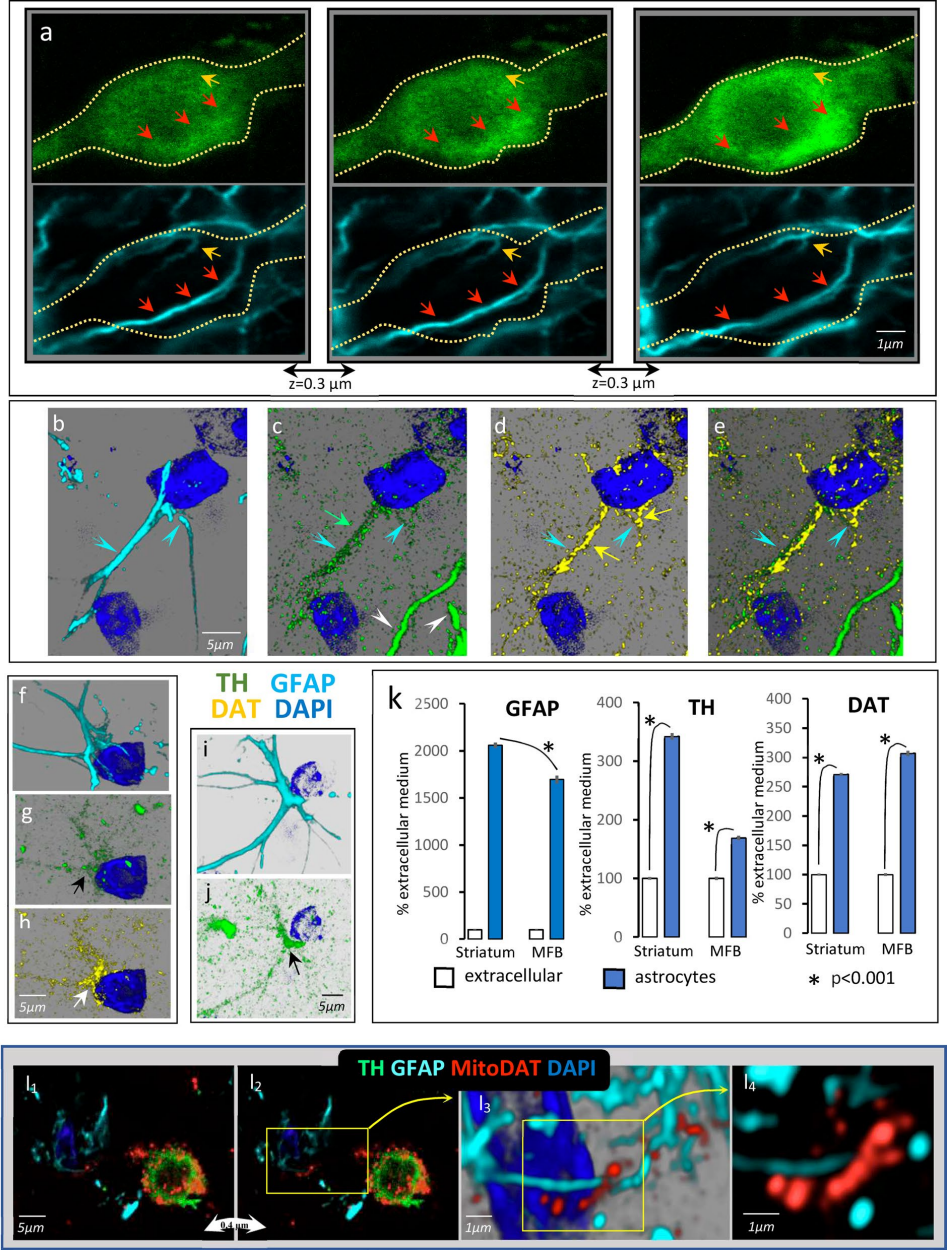
Autophagy of dopaminergic axon ends in nearby astrocytes. **a** Three successive slices (0.3 µm thick) of a spheroid generated from a thickened axon and penetrated by thin (red arrows) and very thin (brown arrows) astrocytic processes (TH in green, GFAP in cyan). **b**–**e** An astrocyte (cyan arrow-head with GFAP in cyan and DAPI in blue) which presented immunoreactivity for TH (green arrow in **c**) and DAT (yellow arrows in **d**) near a degenerating DAergic axon of the MFB (white arrow-head in **c**). **f–h** An astrocyte (cyan in **f**) which presented immunoreactivity for TH (green in **g**) and DAT (yellow in **h**), near a degenerating DAergic spheroid of the MFB (shown at the top-right of **g**). **i, j** Another example of an astrocyte (cyan in **i**) near an MFB spheroid (**j** left side), which accumulated TH immunoreactivity (**j**). **k** The mean ± standard error of the density of GFAP, TH and DAT immunoreactivity (normalized as a percentage of the mean value of the extracellular medium) for MFB spheroids (*n* = 200; *P < 0.001). **l** An example of a spheroid (TH in green) that accumulated DAergic mitochondria, a portion of which was transferred to a nearby astrocyte (cyan). **l**_**1**_, **l**_**2**_ Consecutive 0.4-µm thick slices which accumulated DAergic mitochondria (red). The astrocyte of this example presented some DAergic mitochondria near its nucleus (DAPI in blue) and some of its GFAP filaments. **l**_**3**_ A 3D magnification of the zoomed area in **l**_**2**_. **l**_**4**_ A magnification (0.3 µm thick) of the zoomed area in **l**_**3**_, which presents DAergic mitochondria and GFAP astrocytic filaments located in adjacent areas

Many DAergic spheroids of the MFB were penetrated by astrocytic processes. Figure 6a shows three successive slices (0.3 microns thick) of a thickened axon that was beginning to generate a spheroid, with an already formed typical flocculation region inside (TH in green). This is an example of a spheroid penetrated by astrocytic processes (GFAP in cyan), a spheroid penetrated by both thin (red arrows) and very thin (brown arrows) astrocytic processes (another example is shown in Additional file 8: Video 8). The astrocytic processes (cyan arrowhead in Fig. 6b–e) near the degenerating DAergic axons (white arrowhead in Fig. 6c) showed immunoreactivity for TH (green arrow in Fig. 6c) and DAT (yellow arrows in Fig. 6d), which were very close but did not colocalize in the same region of the astrocytic process (Fig. 6e). The same high immunoreactivity for TH (black arrows in Fig. 6g, j) and DAT (white arrow in Fig. 6h) was found in the somata of astrocytes located in the MFB regions with DAergic spheroids (see also Additional file 9: Video 9). The astrocytes of the MFB regions with DAergic spheroids showed accumulated TH and DAT immunoreactivity (Fig. 6k), even though the MFB astrocytes did not show clear evidence of astrocytosis (the GFAP immunoreactivity of MFB astrocytes was lower than that found in hypertrophic astrocytes of the striatum; Fig. 6k left side).

Studies in the YFP-Mito-DAn mice suggest that mitochondria of damaged axons that were stored in spheroids were later moved to astrocytes. Figure 6l shows an example suggesting that DAergic mitochondria accumulated in spheroids may be transferred to nearby astrocytes. Figure l_1_ and l_2_ represent two consecutive 0.4-µm thick slices showing a TH^+^ spheroid (green) with a typical flocculation area inside and accumulated DAergic mitochondria (red). This spheroid was near an astrocyte (cyan) which had some DAergic fluorescent mitochondria near its nucleus. Figure l_4_ shows a 0.3-µm thick slice of a region of the astrocyte indicated in l_3_, which had DAergic mitochondria and GFAP astrocytic filaments located in adjacent areas. Evidence for the transfer of mitochondria from DAergic spheroids to astrocytes was not clearly found in the case of spheroids generated from MFB axons, even using the Mito-DAT mice. Most astrocytes involved in supporting DAergic axons are located outside the MFB and, as previously commented, many DAergic mitochondria began to be degraded during the generation of spheroids and lost their YFP fluorescence before their arrival at the astrocytes.

**Fig. 7:**
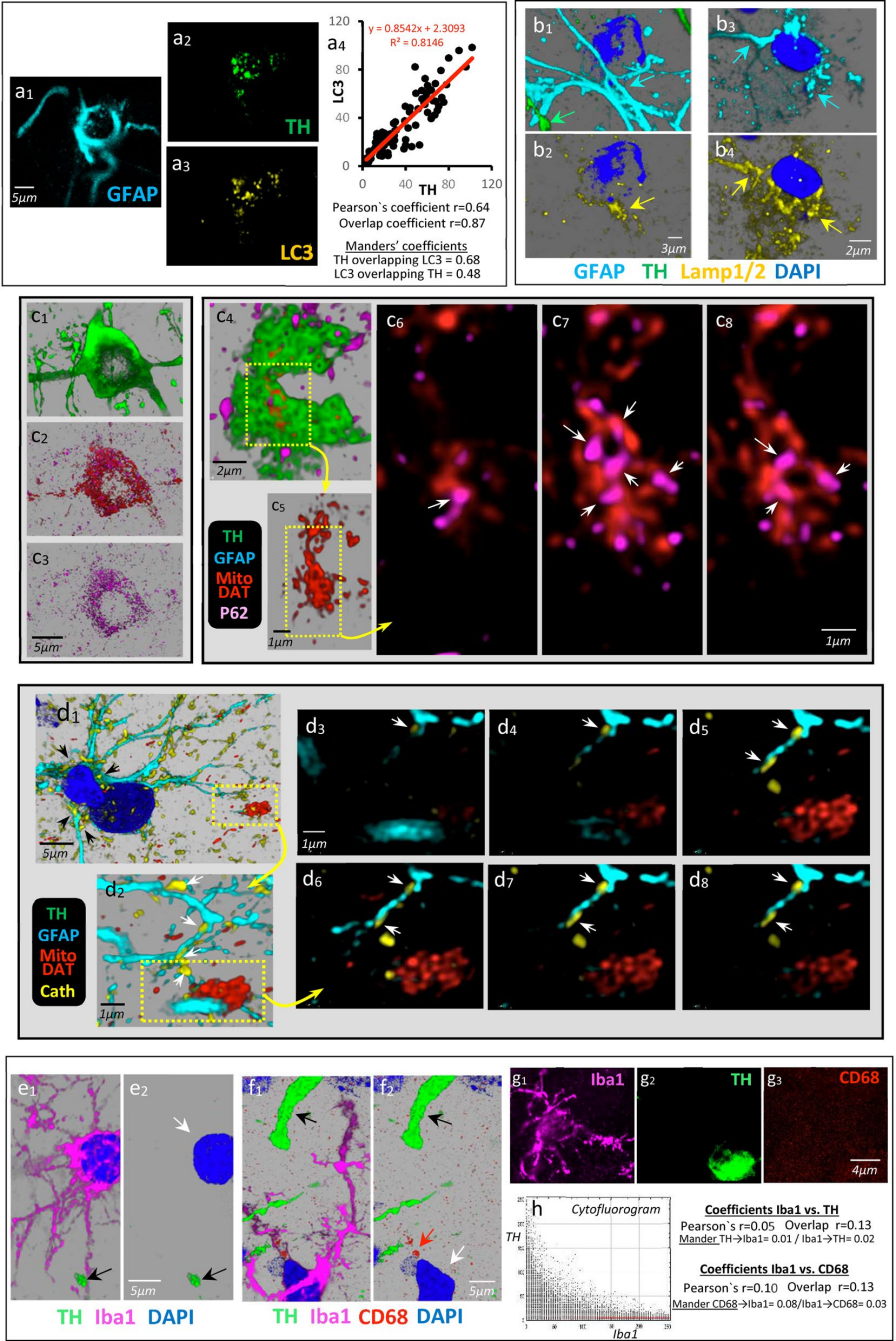
Astrocytes near MFB spheroids present the autophagy mechanisms needed to process the dopaminergic detritus. MFB astrocytes (**a**_**1**_) showed TH (**a**_**2**_) and LC3 (**a**_**3**_) immunoreactivity. **a**_**4**_ The LC3 and TH levels were highly correlated, and Pearson and Manders coefficients showed TH-LC3 colocalization. **b**_**1**_–**b**_**2**_ and **b**_**3**_–**b**_**4**_ show two examples of MFB astrocytes (cyan arrows; GFAP in cyan and DAPI in blue) near degenerating axons and spheroids of the MFB (green arrow in **b**_**1**_) and showing Lamp1 (yellow arrow in **b**_**2**_) and Lamp2 (yellow arrow in **b**_**4**_) immunoreactivity. DAergic cells (**c**_**1**_) of the substantia nigra with MitoDAT mitochondria (**c**_**2**_) expressed P62 (**c**_**3**_), a protein also found in spheroids (**c**_**4**_–**c**_**8**_). **c**_**4**_ A 3D example of a spheroid showing aggregation of mitochondria inside (**c**_**5**_). **c**_**6**_–**c**_**8**_ Consecutive slices of the aggregated mitochondria of **c**_**5**_ connected by P62 protein (white arrows). Cathepsin was found in the somata (black arrows in **d**_**1**_) and processes (white arrows in **d**_**2**_) of astrocytes of the degenerating MFB. **d**_**3**_–**d**_**8**_ Consecutive slices showing that the cathepsin immunoreactivity was particularly high in the astrocytic processes that penetrated or surrounded the mitochondrial aggregations of spheroids but not within the spheroids (white arrows). **e**–**g** Microglial cells (Iba1 in pink and DAPI in blue) located near spheroids or degenerating axons (black arrows) of the MFB neither accumulated TH (green) nor showed CD68 immunoreactivity. **h** TH-Iba1 cytofluorogram, and the Iba1-TH and Iba1-CD68 Pearson and Manders coefficients (*n* = 130) showing no statistical colocalization

The astrocytes with TH immunoreactivity (Fig. 7a_1_) showed immunoreactivity for LC3 in their soma (Fig. 7a_3_), and the TH and LC3 were colocalized in the soma of these astrocytes (Fig. 7a_4_, Additional file 10: Video 10). The astrocyte (cyan arrows in Fig. 7b_1_, b_3_) located near the spheroid (green arrow in Fig. 7b_1_) also showed high immunoreactivity for Lamp1 (yellow arrows in Fig. 7b_2_) and Lamp2 (yellow arrows in Fig. 7b_4_) of lysosomes, while the Lamp1/Lamp2 immunoreactivity was not found in that DAergic spheroids (Fig. 7b_2_). The protein P62 involved in the processing of damaged mitochondria was observed in the spheroid (Fig. 7c). The DAergic cell (Fig. 7c_1_) of the SN with MitoDAT mitochondria (Fig. 7c_2_) expressed P62 (Fig. 7c_3_), a protein also found in spheroids (Fig. 7c_4_–c_8_). Figure 7C_4_ shows an example of a spheroid showing aggregation of mitochondria inside (Fig. 7c_5_). Most of these aggregated mitochondria were connected by P62 (Fig. 7c_6_–c_8_, white arrows). The lysosomal protease cathepsin was observed in the somata (Fig. 7d_1_, black arrows) and processes (Fig. 7d_2_, white arrows) of astrocytes of the degenerating MFB. Puncta of cathepsin immunoreactivity were particularly high in the astrocytic processes that penetrate or surround the mitochondrial aggregations of spheroids but not within spheroids (Fig. 7d_2_–d_7_, white arrows). Taken together, the Lamp1, Lamp2 and cathepsin data suggest that spheroids do not contain lysosomes. Thus, spheroids can generate autophagosomes but do not contain lysosomes, and the autophagy process which starts in spheroids needs lysosomes of the nearby astrocytic processes to proceed.

In addition, microglia/macrophage (Iba1 in Fig. 7e_1_, f_1_) in MFB regions with DAergic spheroids (black arrows in Fig. 7e_1_, e_2_) or thickened TH^+^ degenerating axons (black arrows in Fig. 7f_1_, f_2_) showed no TH immunoreactivity (white arrows in Fig. 7e_2_, f_2_). Microglia (Fig. 7g_1_) near spheroids (Fig. 7g_2_) showed no CD68 immunoreactivity (Fig. 7g_3_). Thus, although some microglial cells presented occasional CD68^+^ lysosomes (Fig. 7f_2_, red arrow), the colocalization studies showed that these cells did not accumulate TH or CD68. Figure 7h shows the TH-Iba1 cytofluorogram, and the Iba1-TH and Iba1-CD68 colocalization coefficients (*n* = 130).

## Discussion

The present work studied the metabolization of axonal debris produced in the MFB by the retrograde degeneration of DAergic nigrostriatal neurons. Proteins and organelles (mitochondria) normally moving along the DAergic axon were accumulated in the degenerating axons and stored in spheroids. The spheroids generated autophagosomes to initiate the macro-autophagy, but this process could not end in these structures since they did not have lysosomes. The thickening axons of degenerating DA cells and spheroids produced by the fracture of these axons were surrounded and/or penetrated by astrocytic processes which moved their DAergic debris to the astrocyte somata for its degradation. Microglial phagocytosis of DAergic debris was not found in the MFB of these animals. Taken together, the present data suggest that the axonal debris produced from retrograde degeneration of DAergic nigrostriatal neurons is stored in spheroids and engulfed by astrocytes, which may prevent the activation of microglial phagocytosis and neuroinflammation that is normally linked to the DA-cell degeneration in PD. The trans-cellular degradation that links the partial autophagy of DAergic debris in spheroids with a later astrocytic phagocytosis (axonal transautophagy) may be critical for preventing PD.

As recently reported, selective degeneration of DAergic innervation of the striatum modifies local astrocytes (e.g. upregulation of GFAP, GS, S100β, NDRG2 and vimentin) but not microglia [7, 27]. The present study shows that this selective loss of DAergic terminals is followed by retrograde DA-cell degeneration which transforms healthy axons (~ 0.5 µm diameter) into thick axons (> 2 µm diameter) that would fracture to produce ball-shaped structures called spheroids (2–9 µm diameter). The spheroids store proteins that normally move anterogradely (TH, DAT, Vmat2 and APP) or retrogradely (GDNF) through healthy axons, thus avoiding their jumbled dispersion across the extracellular space. Ejection of cell detritus to the extracellular medium may activate the microglial phagocytosis, particularly in the case of damaged mitochondria whose components may be highly immunogenic [20, 30–32]. Here, we found that although most of the axonal proteins and mitochondria of the degenerating axons were stored in DAergic spheroids, this accumulation did not occur with all proteins, for example, Syn. Syn is a synaptic protein with intense axonal transfer from the cell somata (where it is synthesized) to the synapse (where it performs its main physiological functions), and which returns from the synapse to the cell somata (where it is degraded in the proteasome). The retention of the axonal traffic of syn, together with the tendency of this protein to build fibrils and aggregates, facilitates the production of somatic Lewy bodies and synaptic neurites that characterize the degenerating DA cells in PD [33, 34]. However, when these cells die, the syn is released to the extracellular medium [35, 36] and absorbed via endocytosis by other cells [37–40], which is consistent with the inability of spheroids to retain the syn found here.

The present data suggest that the proteins retained in degenerating axons and spheroids began but did not finish their metabolization in these structures [41–43]. The finding of clusters of LC3 immunoreactivity suggests the formation of autophagosomes in spheroids. During the formation of autophagosomes, the cytosolic LC3 (LC3-I) is conjugated to phosphatidylethanolamine to form LC3-II, which is specifically targeted to the membrane of nascent autophagosomes (phagophores) [44, 45]. Thus, the production of autophagosomes is associated with accumulation of LC3 immunoreactivity in small cytosolic aggregates that correspond to the fluorescent puncta found here in spheroids with confocal methods [43, 44, 46–50]. The production of autophagosomes was verified by EM studies that identified phagophores and autophagosomes in DAergic spheroids. Thus, the first step of autophagy (generation of autophagosomes) takes place in spheroids. The next step is the fusion of autophagosomes and lysosomes [47], which did not occur in spheroids that neither showed immunoreactivity for Lamp1/Lamp2 or cathepsin (markers of lysosomes) [51, 52] in confocal studies nor showed images compatible to autophagolysosomes in EM studies. These data suggest that the autophagy process which starts in spheroids is incomplete and does not complete the debris metabolization. This should not be surprising because the autophagic process needs energy resources which are probably unavailable in spheroids, and the lysosomes needed for the final degradation of debris are normally located in cell somata rather than in DAergic axons. Astrocytes, and particularly astrocytic processes near spheroids, contained lysosomes needed to complete the degradation of DAergic detritus, suggesting that the lysosomes involved in the autophagy of degenerating DAergic axons are provided by neighboring astrocytes [4].

In addition, the axonal spheroids were surrounded by astrocytic processes which crossed the spheroids from one side to the other. Proteins of DAergic axons not normally observed in astrocytes (TH, DAT) were found in the cytoplasm of astrocytes of the MFB. These proteins were initially found in astrocytic processes and later in the cytosolic regions near the astrocyte nucleus. These data suggest that proteins of degenerating DAergic neurons are transferred to the astrocytic processes and are then displaced to the astrocytic soma. However, it is not possible to rule out that a fraction of the dopaminergic proteins can be released to the extracellular medium and then endocytosed by neighboring astrocytes. The DAergic protein colocalization with LC3 in the somata of astrocytes, where the Lamp1/Lamp2 immunoreactivity was found, indicated a high density of lysosomes. Taken together, the present data provide evidence that the debris originating from the MFB by retrograde degeneration of DAergic axons is processed by a complex mechanism that involves cooperation of the degenerating DAergic axons and the surrounding astrocytes (axonal transautophagy). The degenerating axons and spheroids of the MFB also contained accumulation of DAergic mitochondria. Many mitochondria were grouped inside the spheroid, a clustering that may be induced by the action of the P62 protein, which was often observed between the grouped mitochondria here and has been reported to be involved in mitochondria clustering [53, 54]. A portion of mitochondria of the spheroids were near the autophagosomes, suggesting that mitophagy may begin in these structures. However, the lack of lysosomes (and the break of axons which blocks the transport of damaged mitochondria to the cell somata) prevents the normal mitophagy during the retrograde degeneration of DAergic cells. Instead, the present evidence suggests that the DAergic mitochondria that cannot be transported to the DA-cell somata may be moved to astrocytes to complete their metabolization (transmitophagy), although additional evidence is required to confirm this possibility for mitochondria of the DAergic axons of the MFB. This could be a useful mechanism to prevent activation of neuroinflammation which, in the case of MFB axons, can be a direct threat to the survival of the nigrostriatal DAergic neurons. No evidence of microglial activation or microglial phagocytosis was observed in the MFB region with degenerating DAergic axons. Most microglial cells were located far from the degenerating axons and spheroids, and the few microglial cells whose processes were found near these structures did not engulf DAergic debris. Thus, the present data suggest that transautophagy and transmitophagy mainly involve astrocytes, thus preventing microglial phagocytosis and the triggering of neuroinflammation.

## Conclusions

The axonal transautophagy reported here is a complex mechanism that requires a sequence of events including: (1) storage of DAergic debris within spheroids; (2) arrangement of the intra-spheroidal debris within autophagosomes; (3) approach of astrocytic processes and penetration of the spheroids; (4) transfer of the spheroidal debris to the cytoplasm of astrocytes; and (5) transport of DAergic debris to the cell soma of astrocytes where they may continue their degradation. The DA cells present slow degeneration throughout life, producing axonal debris that needs to be continuously removed. The withdrawal of this debris by microglial phagocytosis could trigger neuroinflammation and facilitate the retrograde degeneration of healthy DAergic axons. The axon-astrocyte transautophagy may be an efficient mechanism to prevent this possibility in normal people, and its failure may be involved in the onset and progression of PD. The failure of this axonal transautophagy could be induced by ageing of astrocytes [3, 55], but also by other mechanisms including accelerated degeneration of DA cells (e.g. caused by environmental neurotoxics, occasional infections, etc.) which produces a large amount of DAergic waste that can no longer be metabolized by astrocytes, resulting in the recruitment of microglia (the “professional” phagocytes) and the activation of neuroinflammation. In summary, the MFB contains a high density of DAergic axons that are close to each other in a small volume. Previous studies have reported that the striatal neuroinflammation produces not only local effects in the striatum but also axonal damage that moves the inflammatory process from the striatum to the SN where degeneration of the somata of DA cells occurs [8, 56]. The axon-astrocyte transautophagy in the MFB may prevent this widespread deterioration, raising a new scenario for the etio-pathogenic control of PD progression.

## Supplementary Information

**Additional file 1: Video 1.** The healthy axons of the MFB (TH in green) accompanied by thin astrocytic process (GFAP in blue) that follows a parallel course to them (DAPI in blue).

**Additional file 2: Video 2.** A degenerating DAergic axon (TH in green) penetrated by an astrocytic process (GFAP in blue).

**Additional file 3: Video 3.** A DAergic **s**pheroid (TH in green) with the internal low-density associated with flocculation and which is surrounded by a small astrocytic process.

**Additional file 4: Video 4.** A DAergic spheroid (TH in green) containing Vmat2 (red) and APP (pink).

**Additional file 5: Video 5.** A DAergic spheroid (TH in green) near astrocytic processes (GFAP in cyan) and containing GDNF (in red).

**Additional file 6: Video 6.** Long mitochondria (YFP fluorescence in red) inside normal axons of the striatum of a YFP-Mito-DAn mouse.

**Additional file 7: Video 7.** A DAergic spheroid (TH in green) accumulating Vmat2 (yellow) and containing many autophagosomes (pink).

**Additional file 8: Video 8.** A DAergic spheroid (TH in green) penetrated by an astrocytic process (cyan) and which accumulates APP immunoreactivity (pink).

**Additional file 9: Video 9.** An MFB astrocyte (GFAP in cyan and DAPI in blue) near degenerating DAergic axons of the MFB (TH in green) and which contains high TH immunoreactivity in its processes.

**Additional file 10: Video 10.** An astrocyte of the MFB (cyan) with colocalization of the TH (green) and LC3 (yellow) immunoreactivity.

## Data Availability

All data generated or analysed during this study are included in this article and supplementary information files.

## Abbreviations

APP: Amyloid precursor protein
CD68: Cluster of differentiation 68
DA: Dopamine
DA-cell: Dopaminergic nigrostriatal neurons
DAT: Dopamine transporter
GFAP: Glial fibrillary acidic protein
Iba1: Ionized calcium binding adaptor molecule 1
Lamp1: Lysosomal-associated membrane protein 1
Lamp2: Lysosomal-associated membrane protein 2
LC3: Microtubule-associated protein 1A/1B-light chain 3
NGS: Normal goat serum
PD: Parkinson’s disease
SN: Substantia nigra
Syn: α-Synuclein
TH: Tyrosine hydroxylase
VMat2: Vesicular monoamine transporter 2
YFP: Yellow fluorescent protein
6-OHDA: 6-Hydroxydopamine
